# Chemical analysis, antioxidant and antimicrobial activities of *Nardostachys jatamansi* essential oil, and computational evaluation of mechanisms

**DOI:** 10.3389/fnut.2026.1764021

**Published:** 2026-03-03

**Authors:** Hang Zhang, Haixia Qin, Jinyao Zha, Yu Zheng, Jing Ji, Rui Chen, Taoshi Liu, Jianming Cheng

**Affiliations:** 1 School of Pharmacy, Nanjing University of Chinese Medicine, Nanjing, China; 2 Jiangsu Province Engineering Research Center of Classical Prescription, Nanjing, China

**Keywords:** antibacterial, antioxidant, GC–MS, molecular docking, *Nardostachys jatamansi* essential oil

## Abstract

**Introduction:**

*Nardostachys jatamansi* DC. is a valued herb in traditional Chinese medicine, historically used to regulate qi flow, alleviate pain, and enhance digestive function. This study aimed to characterize the chemical composition of its essential oil (NJEO) and evaluate its antioxidant and antibacterial activities, complemented by computational analyses to elucidate underlying mechanisms.

**Methods:**

NJEO was extracted via hydrodistillation and analyzed by GC–MS. *In vitro* antioxidant assays (DPPH, ABTS^+^, O_2_•^−^) and antibacterial tests against *Staphylococcus aureus* and *Escherichia coli* were performed. Molecular docking was used to examine binding interactions of major constituents with target proteins.

**Results:**

The extraction yield was 4.28%. GC–MS identified 30 compounds (99.61% of total oil), with terpenes (68.83%) and aromatic compounds (15.01%) as major fractions. NJEO exhibited significant radical scavenging activity, with superoxide scavenging capacity superior to ascorbic acid. It also showed considerable antibacterial activity against both tested strains. Docking analysis revealed that 2,4,5-Trifluoro-3-methoxybenzamide, N-(2,5-dimethoxyphenyl)-, a key constituent, binds strongly to antioxidant and antibacterial targets.

**Conclusion:**

NJEO demonstrates potential as a natural source of antioxidant and antimicrobial agents, with promising applications in pharmaceutical and functional food development.

## 1 Introduction

*N. jatamansi* is an ancient aromatic medicinal plant with a documented application history exceeding 1,200 years, ranking it among the earliest aromatic plants used by humans. It has been extensively employed in traditional medical systems including Ayurveda, Tibetan medicine, and Traditional Chinese Medicine (TCM) for the management of neurological disorders, digestive scomplaints, and cardiovascular conditions (1). In recent years, the growing interest in natural medicines and phytochemicals has positioned *N. jatamansi* as a subject of modern pharmacological research, largely due to its distinctive essential oil and bioactive constituents (2). The distinctiveness of *N. jatamansi* arise not only from its intense aroma, but also from its complex chemical profile and diverse pharmacological activities. Advanced analytical techniques such as gas chromatography–mass spectrometry (GC–MS) and High-Performance Liquid Chromatography (HPLC) have identified numerous key components in NJEO, including jatamansone, *α*-pinene, α-gurjunene, calarene, patchouli alcohol, aristolone, 1,1,7,7a-tetramethyl-1a,2,6,7,7a,7b-hexahydro-1H-cyclopropa[a]naphthalene, (1aR,7R,7aR,7bS)-(+)-1a,2,3,5,6,7,7a,7b-octahydro-1,1,7,7a-tetramethyl-1H-cyclopropa[a]naphthalen-3-one, valeranone, aristolene, *β*-maaliane, *α*-curcumene, and β-patchoulene (3–11). Collectively, these compounds contribute to the plant’s characteristic fragrance and exhibit significant biological activities, highlighting the considerable potential of *N. jatamansi* in neuroprotection, anti-inflammation, antioxidation, and antimicrobial applications (12).

*N. jatamansi,* a member of the Caprifoliaceae family, is a widely used botanical in Tibetan medicine, where its dried roots and rhizomes are the primary medicinal parts. It is known for its functions of regulating qi to relieve pain and relieve depression to strenghen the spleen, and is widely used clinically for bloating, poor appetite, and vomiting (9, 13). Modern pharmacological research has further elucidated its diverse bioactive components—such as essential oil, sesquiterpenes, and lignans—and there broad spectrum of pharmacological effects.

Specifically, valeranone and jatamansone in NJEO exert sedative effects by modulating GABA receptors (14, 15); patchouli alcohol exhibits anti-inflammatory, antibacterial, and antioxidant activities (16–19); *β*-ionone demonstrates antitumor, anti-teratogenic, and antimicrobial properties (20, 21); *α*-pinene possesses antifungal, antimicrobial, and antitumor effects (22–25); and *β*-pinene dispalys antibacterial activity (26–28). Beyond these actions, NJEO also exerts cardiovascular benefits: it stabilizes cardiac rhythm by prolonging the myocardial action potential and inhibiting sodium and potassium channels (29). Additionally, the phenolic components of *N. jatamansi,* such as tannins and flavonoids, mitigate oxidative stress and scavenge free radicals (30–32).

Collectively, these findings indicate that NJEO protects against myocardial ischemia–reperfusion injury (33–35), exhibits robust DPPH free-radical scavenging capacity and reducing power (36–39), and inhibits common pathogens including *Staphylococcus aureus (S. aureus), Escherichia coli (E. coli), Candida albicans Salmonella and Bacillus subtilis* (40–45). Such properties position NJEO as a promising candidate for the development of novel natural antibacterial agents.

While numerous *in vitro* studies have demonstrated the potent antioxidant and broad—spectrum antibacterial activities of NJEO and its major constituents, the precise molecular mechanisms underlying these effects remain only partially understood (46). Conventional pharmacological approaches struggle to delineate the intricate target proteins. This gap in mechanistic insight not only limits our in-depth understanding of NJEO’s antioxidant and antibacterial activities, but also hinders its further development and application.

In recent years, molecular docking-a core technique within computer—aided drug design (CADD) has emerged as a powerful tool for revealing the mechanisms of action of natural products (47). As a computational simulation method, molecular docking aims to predict the optimal binding mode and affinity between small molecules and biomacromolecules in three-dimensional space. It enables the visual depiction of interactions between small molecule and the amino acid residues of target protein, and can rapidly and cost-effectivelly screen lead compounds that bind to specific targets from libraries containing millions to tens of millions of compounds. In doing so, it provides molecular-level theoretical support and mechanistic hypotheses to explain experimental observations (48).

Building on this, the present study integrates *in vitro* bioactivity evaluation, chemical composition analysis, and molecular docking simulations to elucidate the antioxidant and antibacterial molecular mechanisms of NJEO. Key bioactive components will be selected based on their optimal binding energy and subjectes to docking simulations against a panel of target proteins critical to bacterial survival and pathogenicity. This work seeks to theoretically identify potential molecular targets, uncover novel insights into the antibacterial and antioxidant mechanisms of *N. jatamansi*, and lay a solid foundation for the development of novel antibacterial agents derived from natural products.

## 2 Materials and methods

### 2.1 Materials and reagents

Nardostachyos Radix et Rhizoma (*Nardostachys jatamansi DC.*) was procured from Nanjing Heling Pharmaceutical Services Co., Ltd.

*S.aureus* (ATCC 6538) and *E. coli* (ATCC 25922) were obtained from Beijing Beina Chunguang Biotechnology Research Institute.

DPPH and ABTS were sourced from Fujian Feijing Biological Technology Co., Ltd.

Potassium persulfate was purchased from Shaoguan Guangweng Chemical Reagent Co., Ltd.

### 2.2 Volatile oil extraction

Dried and powdered Nardostachyos rhizomes (35 g) were subjected to hydro-distillation for 5 h using a solid-to-liquid ratio of 1:10 (g/mL) (49). The extract was cooled, dehydrated with anhydrous sodium sulfate, and the volatile oil collected and stored at 4 °C in a sealed amber vial until use (50). The extraction yield was calculated as follows:

Extraction yield (%) = V/M × 100%

#### 2.2.1 Chemical composition analysis

A 100 μL aliquot of the NJEO was diluted to 100 mL with n-hexane, mixed thoroughly, and filtered through a 0.22 μm membrane prior to injection (51).

#### 2.2.2 Gas chromatography conditions

Separation was achieved using an Agilent HP-5MS capillary column (15 m × 0.25 mm, 0.25 μm) with helium as carrier gas (1.5 mL/min). Injection was performed in split mode (20, 1) with 1 μL sample at 280 °C. The oven temperature was held at 100 °C for 10 min, raised to 180 °C at 3 °C/min, and held for 5 min. Total run time was 45 min.

#### 2.2.3 Mass spectrometry conditions

EI ionization was applied at 70 eV. The ion source and quadrupole were maintained at 230 °C and 150 °C, respectively. Full-scan acquisition covered m/z 50–500.

#### 2.2.4 Compound identification and quantification

Components were identified by comparison with the NIST11 database and literature reports. Relative contents were determined via peak area normalization.

### 2.3 Antioxidant activity

#### 2.3.1 DPPH radical scavenging assay

NJEO was serially diluted in anhydrous ethanol to prepare 10 working solutions with concentration ranging from 0.039 to 20 μL/mL. AA was dissolved in anhydrous ethanol to serve as the serve as the positive control. An equal volume of each NJEO dilution was mixed with 0.2 mmol/L DPPH ethanol solution. A blank control group and a positive control group were prepared. All mixtures were incubated in the dark at room temperature for 30 min. The absorbance of each samples was measured at 517 nm using a microplate reader (52). A dose–response curve was plotted with NJEO concentration against scavenging rate, and the EC₅₀ value was determined via regression analysis to quantify the free radical scavenging capacity.

#### 2.3.2 Superoxide anion scavenging assay

NJEO was serially diluted in anhydrous ethanol to prepare wprking solutions with concentrations ranging from 5 to 160 μL/mL. AA was dissolved in anhayrous ethanol to serve as the positive reference compound. The reaction mixture was prepared by combining 4.4 mL of 50 mmol/L Tris–HCL buffer (pH 8.2), 0.1 mL of NJEO sample solution, and 0.1 mL of 3 mmol/L pyrogallol solution. The mixture was incubated at 25 °C for 4 min, after which the reaction was terminated by adding concentrated hydrochloric acid (HCl). A blank control and positive control were also prepared and treated identically. The absorbance of each mixture was measured at 325 nm (53). The superoxide anion scavenging rate was calculated, and EC_50_ value was determined from the dose–response curve to quantify the scavenging capacity.

#### 2.3.3 ABTS^+^ radical scavenging assay

The ABTS^+^ stock solution was prepared by reacting 7 mmol/L ABTS^+^ with 2.45 mmol/L potassium persulfate, followed by incubation in the dark for 12–16 h before use. NJEO was seroally diluted in anhydrous ethanol to prepare working solutions with concentrations ranging from (0.078—40 μL/mL). AA was dissolved in anhydrous ethanol to serve as the positive control. The ABTS^+^ stock solution was diluted with anhydrous ethanol to prepare the working solution (adjust to an absorbance of 0.70 ± 0.02 at 734 nm). Each reaction mixture was prepared by combining 0.4 mL of NJEO sample solution with 3.6 mL of ABTS^+^ working solution. A blank control and positive were also prepared. All mixtures were incubated at room temperature for 6–10 min. The absorbance of each mixture was measured at 734 nm (15). The ABTS^+^ radical scavenging rate was calculated, and the EC₅₀ value was determined from the log dose-effect curve to quantify the scavenging capacity.

### 2.4 Antibacterial activity assay

#### 2.4.1 Determination of EC₅₀

NJEO was dissolved in 0.5% dimethyl sulfoxide (DMSO), sonicated at 800 W for 10 min, and diluted with phosphate-buffered saline (PBS) to prepare a 200 μL/mL stock solution. Twofold serial dilutions were performed to obtain 10 working concertration ranging from 0.09 to 400 μL/mL. Vancomycin was used as the positive control, and all experiments were performed in triplicate for reliability. The agar dilution method was employed in a 96-well plate format. The following groups were set up blank control (medium only), negative control (bacteria suspension without NJEO), positive control (bacterial suspension treated with vancomycin), and experimental groups (bacterial suspension with NJEO) at different concentrations. All plates were incubated at 37 °C for 16–24 h. The absorbance of each well was measured at 600 nm to assess bacterial growth (16, 54). The inhibition rate was calculated as:

Inhibition rate (%) = [1−(OD_sanmple_ − OD_blank_)/OD_negative_] × 100%

The EC₅₀ value was determined from the regression curve of log NJEO concentration versus inhibition rate.

#### 2.4.2 Disk diffusion assay

NJEO was diluted with sterile PBS to prepare working solutions of 25, 50, and 100% (v/v). Vancomycin (Van) and Ciprofloxacin (CIP) were dissolved in PBS to prepare positive control solutions (25–100 μg/mL). Sterile PBS was used as the negative control. Sterile paper disks (5 mm diameter) were used as the carrier for samples and controls. A 10 μL aliquot of each NJEO dilution, positive control solution, or negative control (PBS) was applied to the sterile paper disks. The disks were placed on Mueller- Hinton agar plates inoculated with the test bacterial suspension. All plates were incubated at 37 °C for 24 h (55). The diameter of the inhibition zone (including the 5 mm diameter of the paper disk) was measured for each disk (56). All tests were performed in triplicate to ensure reliability.

### 2.5 Molecular docking

Molecular docking was performed to investigate the binding mechanisms of bioactive compounds from Nardostachyos volatile oil with target proteins associated with antioxidant and antibacterial activities. *L. sanfranciscensis* Nox (PDB:2CDU), *S. aureus* TyrRS (PDB:1JIJ), and *E. coli* FabB (PDB:1FJ4) were obtained from the RCSB PDB. Proteins were prepared by removing non-essential water molecules and ligands using *Discovery Studio.* Ligand structures were retrieved from *PubChem*, converted to PDB format, and energy-minimized with *PyMOL*. Docking simulations were carried out using *AMDock* to estimate binding free energy (Δ*G*). The top three compounds exhibiting the strongest binding stability were further analyzed for specific interactions with the target proteins (57, 58).

### 2.6 Statistical analysis

All *in vitro* antioxidant assays were independently repeated three times (*n* = 3). Data are presented as mean ± standard deviation. The EC₅₀ values and the corresponding regression coefficients (R^2^) were calculated by nonlinear regression using a Logistic fit model in Origin 2024.

## 3 Results

All experiment were independently repeated three times (*n* = 3), and the results were expressed as mean ± standard deviation (SD).

### 3.1 Chemical composition analysis of NJEO

**Figure 1:**
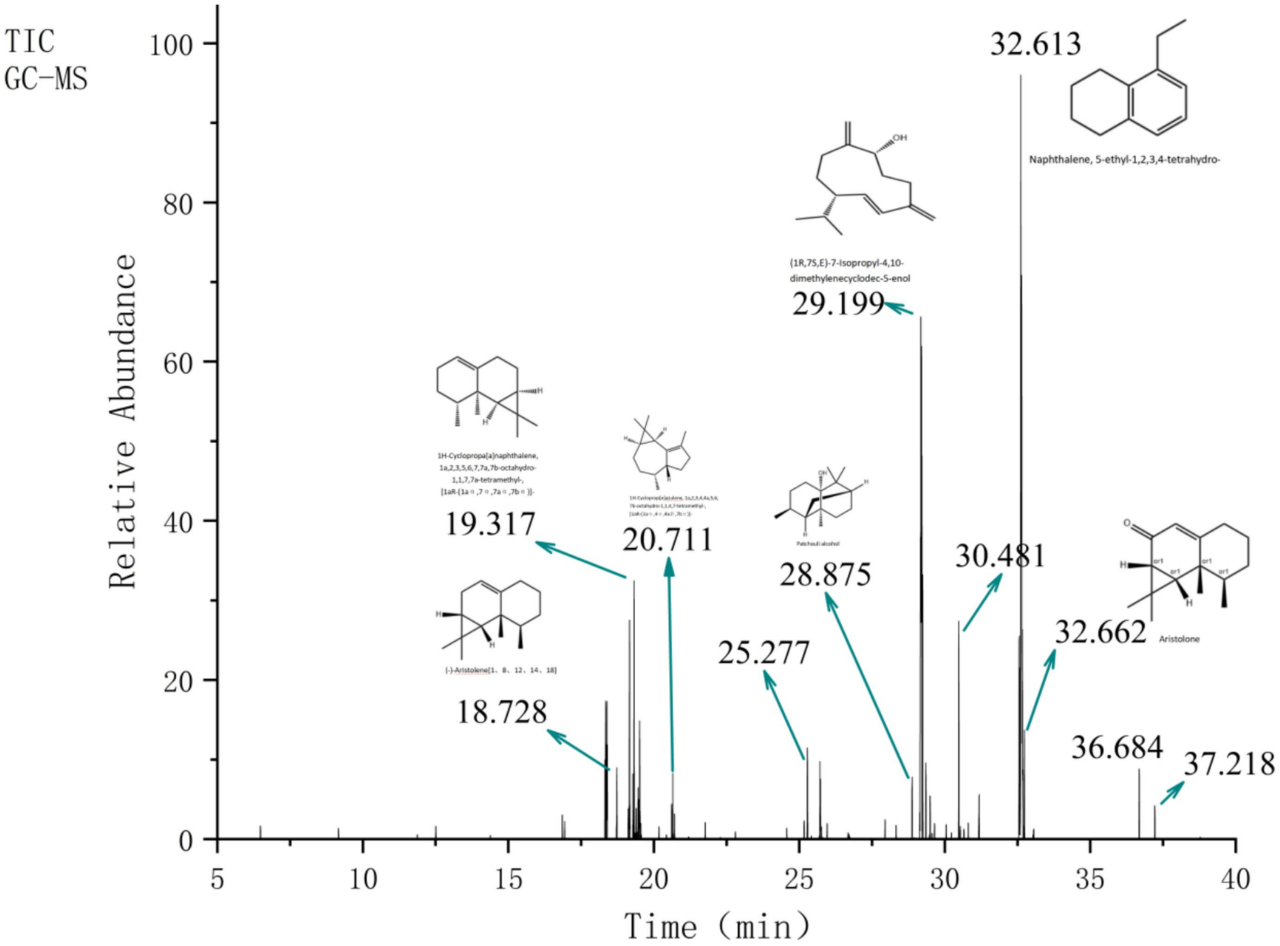
TIC of NJEO obtained ny GC–MS analysis.

Based on a comprehensive consideration of raw material characteristics, extraction cost, and method simplicity, NJEO was extracted by steam distillation in this study, yielding 4.28% (v/w) based on the dry weight of the plant material. A total of 30 compounds were identified by GC–MS analysis, accounting for 99.61% of the total oil composition (Table 1; Figure 1). NJEO mainly consisted of terpenoids and aromatic compounds. Major constituents included cis-Calamenene (18.81%), (1R,7S, E)-7-isopropyl-4,10-dimethylenecyclodec-5-enol (17.48%), Ledene oxide-(II) (14.79%), Naphthalene, 5-ethyl-1,2,3,4-tetrahydro- (12.87%), 1,1,7,7a-tetramethyl-1a,2,6,7,7a,7b-hexahydro-1H-cyclopropa[a]naphthalene (3.82%), aristolone (3.81%), and 1,3-butanedione, 2-[[4-(diethylamino)-2-methylphenyl]imino]-1-phenyl- (3.16%). Minor constituents, each comprising less than 3% of the NJEO, included N-arachidoyl-5-hydroxytryptamine (2.96%), Naphthalene, 1,2,3,4-tetrahydro-1,1,6-trimethyl- (2.14%), (E)-2-((8R,8aS)-8,8a-Dimethyl-3,4,6,7,8,8a-hexahydronaphthalen-2(1H)-ylidene)propyl formate(2.04%), and 2-(4a,8-Dimethyl-2,3,4,5,6,8a-hexahydro-1H-naphthalen-2-yl)propan-2-ol (1.44%).

**Table 1:** Chemical composition of NJEO as identified by GC–MS analysis.

No	RT/min	Area (%)	Intensity	CAS	Identified results	Formula	Compound class
1	18.321	0.41	7908.72	—	—	—	—
2	18.343	1.07	13835.55	469-92-1	3a,7-Methano-3aH-cyclopentacyclooctene, 1,4,5,6,7,8,9,9a-octahydro-1,1,7-trimethyl-, [3aR-(3aα,7α,9aβ)]-	C_15_H_24_	Cyclopropane Derivatives
3	18.386	2.73	13814.43	—	—	—	—
4	18.404	0.66	9413.13	—	—	—	—
5	18.728	1.11	7126.31	6831-16-9	(−)-Aristolene[1, 8, 12, 14, 18]	C_15_H_24_	Terpenes
6	19.122	0.41	3039.37	—	2H-1,3,4-Oxadiazin-2-one, 3,6-dihydro-5,6-dimethyl-6-phenyl-	C_11_H_12_N_2_O_2_	Heterocyclic Compounds
7	19.166	3.82	21970.37	154098-14-3	1,1,7,7a-Tetramethyl-1a,2,6,7,7a,7b-hexahydro-1H-cyclopropa[a]naphthalene	C_15_H_22_	Terpenes
8	19.276	0.30	6554.19	75125-35-8	9,11-Octadecadiynoic acid, 8-oxo-, methyl ester	—	Fatty Acid Derivatives
9	19.317	1.36	25939.25	17334-55-3	1H-Cyclopropa[a]naphthalene, 1a,2,3,5,6,7,7a,7b-octahydro-1,1,7,7a-tetramethyl-, [1aR-(1aα,7α,7aα,7bα)]-	—	Terpenes
10	19.456	0.91	5046.06	—	(2S,4aR,8aR)-4a,8-Dimethyl-2-(prop-1-en-2-yl)-1,2,3,4,4a,5,6,8a-octahydronaphthalene	—	Terpenes
11	19.512	2.04	11751.91	352,457–47-7	(E)-2-((8R,8aS)-8,8a-Dimethyl-3,4,6,7,8,8a-hexahydronaphthalen-2(1H)-ylidene)propyl formate	C_15_H_24_	Terpenes
12	20.616	0.43	3474.28	—	2,4,5-Trifluoro-3-methoxybenzamide, N-(2,5-dimethoxyphenyl)-	C_16_H_14_F_2_NO_4_	Heterocyclic Compounds
13	20.711	0.32	2502.31	489-40-7	1H-Cycloprop[e]azulene, 1a,2,3,4,4a,5,6,7b-octahydro-1,1,4,7-tetramethyl-, [1aR-(1aα,4α,4aβ,7bα)]-	C_15_H_24_	Terpenes
14	25.277	1.44	9043.62	—	2-(4a,8-Dimethyl-2,3,4,5,6,8a-hexahydro-1H-naphthalen-2-yl)propan-2-ol	C15H26O	Terpenes
15	25.710	0.75	7684.96	88395-46-4	Isospathulenol	C_15_H_24_O	Terpenes
16	28.875	0.79	6228.86	5986-55-0	Patchouli alcohol	C_15_H_26_O	Terpenes
17	29.177	14.79	52466	—	Ledene oxide-(II)	C_15_H_24_O	Terpenes
18	29.199	17.48	49513.94	81968-62-9	(1R,7S, E)-7-Isopropyl-4,10-dimethylenecyclodec-5-enol	C_15_H_24_O	Terpenes
19	29.350	0.95	7577.74	—	Glycine, N-ethyl-N-(2-methoxyethoxycarbonyl)-, ethyl ester	C_10_H_19_NO_5_	Amino Acid Derivatives
20	29.497	0.54	4278.02	39,007-93-7	Sesquirosefuran	C_15_H_22_O	Terpenes
21	30.481	3.16	21872.80	—	1,3-butanedione, 2-[[4-(diethylamino)-2-methylphenyl]imino]-1-phenyl-	C_21_H_24_N_2_O_2_	Others
22	31.180	0.89	4479.05	57040-47-8	1,3-Di(propen-1-yl)adamantane	C_16_H_24_	Others
23	32.557	2.96	20384.23	21249-34-3	N-Arachidoyl-5-hydroxytryptamine	C_30_H_50_N_2_O_2_	Others
24	32.576	2.14	17170.46	475-03-6	Naphthalene, 1,2,3,4-tetrahydro-1,1,6-trimethyl-	C_13_H_18_	Aromatic Compounds
25	32.613	12.87	76756.73	42775-75-7	Naphthalene, 5-ethyl-1,2,3,4-tetrahydro-	C_12_H_16_	Aromatic Compounds
26	32.642	18.81	56634.4	72937-55-4	cis-Calamenene	C_15_H_22_	Terpenes
27	32.662	3.81	21031.26	6831-17-0	Aristolone	C_15_H_22_O	Terpenes
28	32.732	1.40	10928.81	114339-93-4	(1aR,7R,7aR,7bS)-(+)-1a,2,3,5,6,7,7a,7b-Octahydro-1,1,7,7a-tetramethyl-1H-cyclopropa[a]naphthalen-3-one	C_15_H_22_O	Terpenes
29	36.684	0.87	6957.37	31575-41-4	Bicyclo [2.2.2]octa-2,5-diene, 1,4,5,7,7,8,8-heptafluoro-2,3-dimethyl-	C_10_H_7_F_7_	Others
30	37.218	0.41	3289.49	—	Alanine, N-methyl-N-(2-methoxyethoxycarbonyl)-, octadecyl ester	C_26_H_5_1NO_5_	Amino Acid Derivatives
	Totally Identified	99.61					

### 3.2 Antioxidant activity assays

*In vitro* antioxidant assays revealed that NJEO exhibited significant concentration-dependent radical-scavenging activity across three distinct assay systems.

As presented in Table 2, the Half-Maximal Effective Concentration (EC₅₀) of NJEO varied notably among the different assays. NJEO demonstrated the strongest activity against DPPH radicals, with an EC_50_ of 1.53 ± 0.19 μL/mL. Its activity against ABTS^+^ radicals was moderate, with an EC_50_ of 3.70 ± 0.49 μL/mL.

**Table 2:** Comparative antioxidant activity of Nardostachyos volatile oil and ascorbic acid.

Assay	Volatile oil (μL/mL)	R^2^	Ascorbic acid (μg/mL)	R^2^
DPPH-EC₅₀	1.53 ± 0.19	0.991	0.67 ± 0.07	0.991
ABTS^+^-EC₅₀	3.70 ± 0.49	0.989	3.70 ± 0.49	0.989
O₂•^−^-EC₅₀	8.11 ± 0.75	0.990	20.89 ± 5.09	0.956

Data are presented as mean ± SD (*n* = 3).

A particularly noteworthy observation was its performance against O₂•^−^. Although the EC_50_ was higher (8.11 ± 0.75 μL/mL), this value it was approximately 2.5 times more potent than that of the reference antioxidant AA (EC_50_ = 20.89 ± 5.09 μg/mL)in the same assay, suggesting that NJEO may possess a selective or enhanced scavenging capacity for scavenging O₂•^−^.

Furthermore, All dose–response curves displayed strong linearity, with regression coefficients (R^2^) for NJEO exceeding 0.95 (Table 2), further supporting a consistent and predictable concentration-dependent response. The relatively higher data variability observed in the O₂•^−^ assay for both NJEO and AA is likely attribute to the inherent methodological variability of this specific assay system.

### 3.3 Antibacterial activity of NJEO

The antibacterial activity of NJEO against representative Gram-positive (*S. aureus*) and Gram-negative (*E. coli*) strains was evaluated using disk diffusion and broth microdilution assays, with results compared to reference antibiotics (Table 3).

**Table 3:** Comparatibe antibacterial activity of NJEO and reference antibiotics.

Title	DIZ* (mm)		EC_50_ (μl/mL)	
	*S. aureus*	*E. coli*	*S. aureus*	*E. coli*
NJEO	11.17 ± 0.51	7.40 ± 0.42	14.70 ± 2.55	20.09 ± 1.31
	7.33 ± 0.20	5.46 ± 0.45		
	2.33 ± 0.24	1.29 ± 0.43		
Van	4.00 ± 0.70	—	2.27 ± 0.21	—
	2.83 ± 0.74	—		
	—	—		
CIP	—	15.50 ± 0.07	—	0.02 ± 0.00
	—	12.43 ± 0.24		
	—	7.78 ± 0.61		

*DIZ: Diameter of inhibition zone. Values are presented as mean ± SD. NJEO was tested at concentrations of 100, 50, and 25% (v/v).

CIP: Ciprofloxacin. Van and CIP were tested at 100, 50, and 25 μg/mL.

Data are presented as mean ± SD (*n* = 3).

—: Not tested or no detectable activity.

A clear dose-dependent inhibitory effect was observed in the disk diffusion assay (Table 3). Against *S. aureus*, NJEO produced inhibition zones of 2.33 ± 0.24 mm, 7.33 ± 0.20 mm, and 11.17 ± 0.51 mm at concentrations of 25, 50, and 100%, respectively. In contrast, its activity against *E.coli* was consistently weaker at the same concentrations, with corresponding zones of only 1.29 ± 0.43 mm, 5.46 ± 0.45 mm, and 7.40 ± 0.42 mm. This trend indicates a pronpunced selectivity of NJEO toward the Gram-positive bacterium. The broth microdilution assay quantitatively confirmed this selectivity (Table 3). The EC_50_ against *S. aureus* was 14.70 ± 2.55 μL/mL which was approximately 1.4 times lower than the EC_50_ against *E. coli* (20.09 ± 1.31 μL/mL), demonstrating stronger bacterical or bacteriostatic potency against the Gram-positive strain (Supplementary Figure 1).

Comparative analysis with standard antibiotics revealed distinct activity profiles, the potency of NJEO, as reflected by its EC_50_ values, was considerably lower than that of the specialized antibiotics Van (for *S. aureus*) and CIP (for *E.coli*). However, a notable finding emerged from the disk diffusion results at the highest concentration (100%): the inhibition zone formed by NJEO against *S. aureus* (11.17 ± 0.51 mm) was substantially larger than that produced by Van at its tested concentration(s). This suggests that while NJEO’s purified active components may be less potent, its crude extract at high concentration can exert a broad and physically substantial zone of inhibition, possibily involving multi-component synergistic or other mechanisms beyond direct molecular targeting.

The relatively larger standard deviation associated with the EC_50_ for *S. aureus* (± 2.55 μL/mL) compared to that for *E. coli* (±1.31 μL/mL) may point to greater variability in susceptibility among replicates or strains of the Gram-positive bacterium to NJEO’s action.

### 3.4 Molecular docking

Molecular docking results demonstrated varying binding potentials of the selected compounds against three key bacterial targets. A notable observation was consistently strong binding affinity of conpound 15 across all targets, with Glide G-scores of −8.6 kcal/mol for *L. sanfranciscensis* Nox, −9.2 kcal/mol for *S. aureus* TyrRS and −8.8 kcal/mol for *E. coli* FabB.

**Figure 2:**
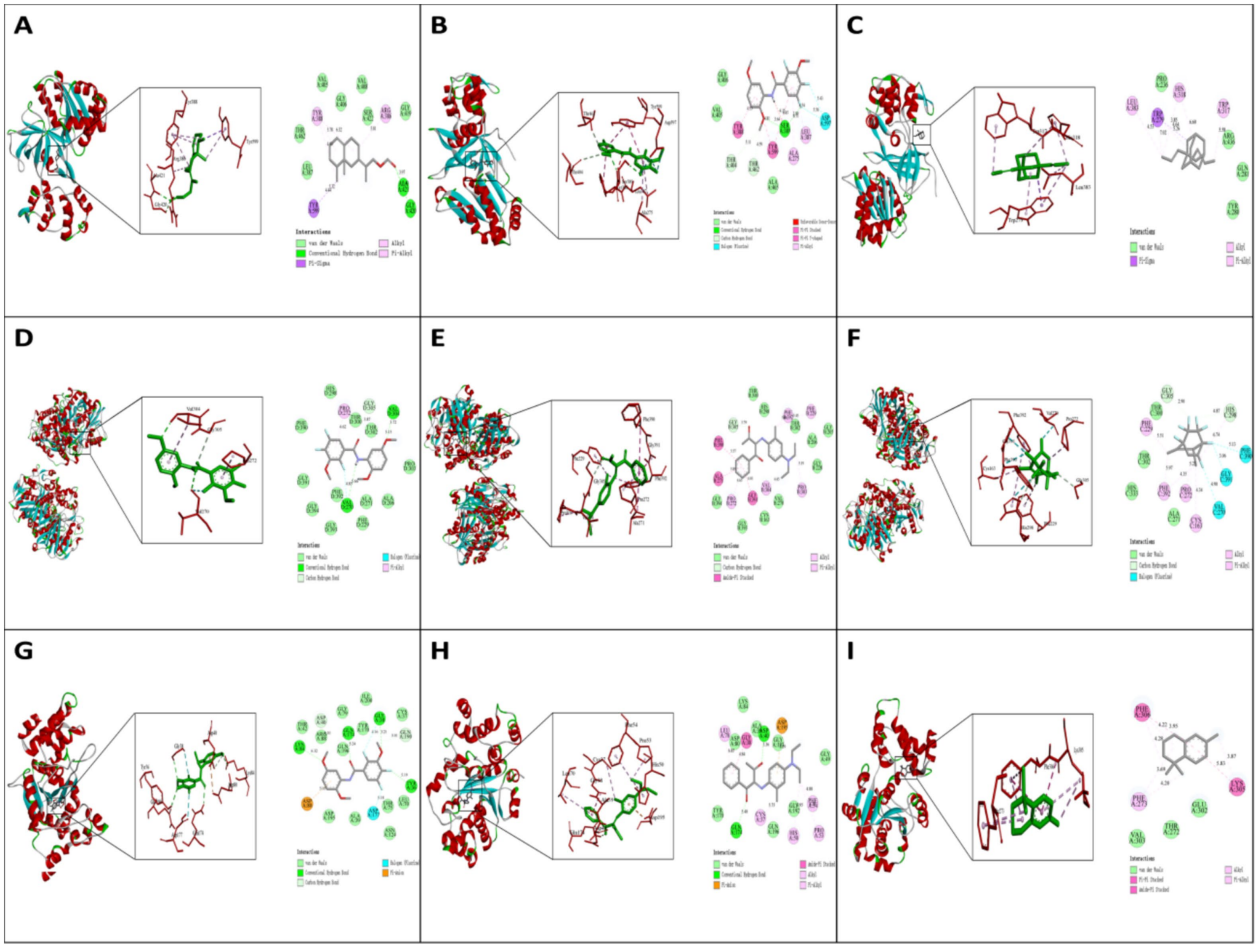
Molecular docking models of NJEO constituents with key target proteins (*S. aureus* TyrRS, *E. coli* FabB, and *L. sanfranciscensis* Nox).

Further structural analysis revealed distinct interaction patterns for compound 15 with each target, which may explain its inhibitory potential. In *S. aureus* TyrRS, it formed hydrogen bonds with Gly38 and Gln190; in *E. coli* FabB, key interactions occurred with Val 270 and Val304; while in *L. sanfranciscensis* Nox, binding was stabilized primarily through a hydrogen bond with Ser389. These specific interactions suggest different inhibitory mechanisms for each target system (Figure 2).

An interesting comparative trend was observed for compound 4, which showed substantial binding to both *S. aureus* TyrRS (−8.5 kcal/mol) and *E. coli* FabB (−9.2 kcal/mol) but significantly weaker activity against *L. sanfranciscensis* Nox (Table 4). This selectivith pattern may indicate shared structural features or binding modes between the two former targets. Overall, these docking studies identify several promising lead compounds, with compound 15 emerging as a particularly interesting candidate for further development as a potential broad-spectrum inhibitor.

**Table 4:** Multi-target docking against *E. Coli* FabB, *S. aureus* TyrRs and *L. sanfranciscensis* nox identifies synergistic polypharmacological compounds in *Nardostachyos jatamansi.*

NO	Molecules	2CDU*	1JIJ	1FJ4
		kcal/mol	kcal/mol	kcal/mol
1	1,1,7,7a-Tetramethyl-1a,2,6,7,7a,7b-hexahydro-1H-cyclopropa[a]naphthalene	−6.7	−6	−6.9
2	Ledene oxide-(II)	−6.5	−6.3	−5.6
3	(1R,7S,E)-7-Isopropyl-4,10-dimethylenecyclodec-5-enol	−6.6	−6.5	−7.7
4	1,3-butanedione, 2-[[4-(diethylamino)-2-methylphenyl]imino]-1-phenyl-	−7.2	−8.5	−9.2
5	Naphthalene, 5-ethyl-1,2,3,4-tetrahydro-	−6.8	−7.3	−7.2
6	cis-Calamenene	−7.3	−7.1	−8.1
7	Aristolone	−6.5	−6.3	−6.4
8	3a,7-Methano-3aH-cyclopentacyclooctene, 1,4,5,6,7,8,9,9a-octahydro-1,1,7-trimethyl-, [3aR-(3aα,7α,9aβ)]-	−6.8	−6.2	−6.8
9	(−)-Aristolene	−6.4	−5.4	−6.5
10	2H-1,3,4-Oxadiazin-2-one, 3,6-dihydro-5,6-dimethyl-6-phenyl-	−6.8	−7.1	−8.3
11	9,11-Octadecadiynoic acid, 8-oxo-, methyl ester	−5.7	−6.5	−7.1
12	1H-Cyclopropa[a]naphthalene, 1a,2,3,5,6,7,7a,7b-octahydro-1,1,7,7a-tetramethyl-, [1aR-(1aα,7α,7aα,7bα)]-	−6.6	−5.6	−7.2
13	(2S,4aR,8aR)-4a,8-Dimethyl-2-(prop-1-en-2-yl)-1,2,3,4,4a,5,6,8a-octahydronaphthalene	−6.6	−5.9	−7.4
14	(E)-2-((8R,8aS)-8,8a-Dimethyl-3,4,6,7,8,8a-hexahydronaphthalen-2(1H)-ylidene)propyl formate	−7.3	−6.5	−6.7
15	2,4,5-Trifluoro-3-methoxybenzamide, N-(2,5-dimethoxyphenyl)-	−8.6	−9.2	−8.8
16	1H-Cycloprop[e]azulene, 1a,2,3,4,4a,5,6,7b-octahydro-1,1,4,7-tetramethyl-, [1aR-(1aα,4α,4aβ,7bα)]-	−7.1	−5.9	−6.2
17	2-(4a,8-Dimethyl-2,3,4,5,6,8a-hexahydro-1H-naphthalen-2-yl)propan-2-ol	−6.7	−6.7	−7.1
18	Isospathulenol	−6.8	−6.9	−7.6
19	Patchouli alcohol	−6.6	−6.1	−5.9
20	Glycine, N-ethyl-N-(2-methoxyethoxycarbonyl)-, ethyl ester	−4.9	−5.8	−6.1
21	Sesquirosefuran	−6.6	−6.8	−7.9
22	1,3-Di(propen-1-yl)adamantane	−7.3	−6.5	−8.2
23	N-Arachidoyl-5-hydroxytryptamine	−6.7	−7.1	−7.1
24	Naphthalene, 1,2,3,4-tetrahydro-1,1,6-trimethyl-	−7	−7.6	−7.6
25	(1aR,7R,7aR,7bS)-(+)-1a,2,3,5,6,7,7a,7b-Octahydro-1,1,7,7a-tetramethyl-1H-cyclopropa[a]naphthalen-3-one	−6.6	−7.5	−7
26	Bicyclo[2.2.2]octa-2,5-diene, 1,4,5,7,7,8,8-heptafluoro-2,3-dimethyl-	−7.1	−7	−8.5
27	Alanine, N-methyl-N-(2-methoxyethoxycarbonyl)-, octadecyl ester	−5.6	−6	−6

*2CDU: NADPH Oxidase (*L. sanfranciscensis*).

1JIJ: tyrosyl-tRNA synthetase (*S. aureus*); 1FJ4: β-ketoacyl-ACP synthase I (FabB) (*E. coli*).

NO: In the molecular docking studies, the compounds are referred to by their corresponding numbers as shown in Table 4.

## 4 Discussion

### 4.1 Chemical composition analysis of NJEO

Essential oils (EOs) are complex volatile mixtures obtained from plant raw materials through various extraction techniques. The selected extraction method significantly influences the chemical composition, yield, and biological activity of the EO (59). Steam distillation (SD) is the most widely used method for extracting volatile oils. Due to its simple equipment, convenient operation, low cost, ease of scale-up, environmental friendliness, and high safety, it is extensively employed in laboratory settings (59). The main difference between co-hydrodistillation (CHD) and SD lies in the plant material being fully immersed in water. This method is suitable for powdered or easily agglomerated materials, ensuring uniform heating and thereby improving extraction efficiency (60). Supercritical CO_2_ extraction (SCE) and continuous phase-transition extraction (CPTE) are widely used in natural product extraction due to their strong extraction capability, high yield, absence of solvent residues, and large processing capacity (61). CPTE utilizes changes in internal system pressure and temperature to drive the liquid–gas phase transition cycle of the solvent. It maximizes energy utilization, enhances mass transfer efficiency, achieves high solvent recovery with low loss, and offers good operational flexibility and adaptability, though it involves high technical complexity and cost (59). Enzyme-assisted extraction (EAE) can significantly improve extraction yield and operate under mild conditions, which helps protect active components. This method is primarily suitable for plant materials with dense cell wall structures, target components sensitive to heat or chemical environments, and applications requiring high product purity and safety (62).

The chemical composition of NJEO in the present study shares a fundamental similarity with other medicinal essential oils, such as *Asarum* oil, in being predominantly composed of terpenoids and aromatic compounds, which are widely recognized for their broad-spectrum biological activities including antimicrobial and antioxidant effects (15).

However, notable quantitative differences exist when compared to earlier reports on *N. jatamansi* oils. For instance, the relative abundances of key constituents such as (−)-Aristolene and Patchouli alcohol in our sample differ from those documented in previous studies (10, 18). These variations likely stem from well-known influencing factors including geographical origin, plant chemotype, harvest conditions, and notable, the extraction method employed.

The significance of these findings lies in establishing a concrete link between a specific chemotypic profile of NJEO and its observed biological activities. Rather than treating *N. jatamansi* oil as a uniform entity, this study highlighs its inherent chemical diversity and underscores how specific compositional signatures-such as the one characterized here-ccorrelate with its functional performance in antioxidant and antibacterial assays.

This chemotype-activity relationship not only supports the traditional use of NJEO but also provides a more reproducible basis for its potential applications in natural preservation and complementary antimicrobial strategies (50).

### 4.2 Antioxidant activity assays

Antioxidant capacity represents a critical pharmacological property in developing natural products for managing oxidative stress-related pathologies. The overproduction of free radicals can induce lipid peroxidation, protein dysfunction, and DNA damage, thereby accelerating aging and promoting chronic disease (63).

When compared to existing studies on *N. jatamansi* this results show both consistencies and instructive variations. The scavenging activity against O₂•^−^ observed in this study aligns closely with the trend reported by Pathak, S. et al. (58), reinforcing the reliability of NJEO in mitigating this physiologically relevant radical species. However, disparities emerged in the DPPH and ABTS^+^ assay trends relative to the same literature (58). These differences likely stem not from contradictory findings but from methodological variability, particularly in solvent systems, which are known to differentially influence the solubility, stability, and electron-transfer capacity of antioxidant constituents in complex EO matrices.

Beyond direct radical quenching, the antioxidant activity of NJEO may operate through a complementary dual mechanism. First, direct neutralization is facilitated by hydrogen or electron donation from its major chemical classes-terpenoids (68.83%) and aromatic compounds (15.01%). Second, indirect cytoprotection may occur via activation of the Nrf2-ARE signaling pathway, leading to the upregulation of endogenous antioxidant enzymes such as SOD and GPx (59, 60). These complementary pathways likely operate cooperatively, forming the foundation for NJEO’s notable antioxidant efficacy.

These findings extend previous reports by not only confirming antioxidant activity but also lingking it to a defined chemical composition and proposing integrated mechanisms. The convergence of direct chemical defense and potential pathway modulation underscores the multifaceted antioxidant character of NJEO. This mechanistic insight enhances its scientific relevance for applications in natural preservative systems or as adjunct in oxidative stress—related therapies, while also highlighting the need for standardized extraction and evaluation protocols to ensure reproducible bioactivity across studies.

### 4.3 Antibacterial activity of NJEO

The broad-spectrum antibacterial activity observed for NJEO corroborates the traditional use of *N. jatamansi* and aligns with the documented bioactivity of terpenoid-rich essential oils (61). Therefore, the antibacterial effect of NJEO is not due to a single compound but rather results from the synergistic “multi-target and multi-mechanism” effect produced by there active terpenoids in combination with other minor constituents.

A critical analysis suggests a multi-mechanistic framework to explain its efficacy. First, the lipophilic nature of its major terpenoid classes supports a well-established mechanism of membrane destabilization. Second, the notable selectivity against Gram-positive bacteria, particularly *S. aureus*, hints at additional target-specific interactions beyond general menbrane disruption. This selectivity finds a plausible explanation in the computational studies, which predicted high-affinity binding of representative NJEO—like compounds to key enzymes essential for bacterial viability, such as TyrRS. This *in silico* evidence provides a novel mechanistic hypothesis connecting the oil’s chemical complexity to a potential mode of action involving critical enzyme inhibition.

When contextualized within existing literature, the result reinforce the understanding that the antibacterial potency of NJEO is inherently variable, influenced by factors such as plant chemotype and processing methods (62). The novelty of this work lies not in reporting another instance of antimicrobial activity, but in integrating bioassay data with computational docking to propose a specific, testable mechanism-enzyme inhibition-that could account for its observed Gram-positive selectivity. This approach shifts the narrative from phenomenological reporting toward mechanistic inquiry, strengthening the scientific rationale for exploring NJEO in strategies against resistant Gram-positive infections.

### 4.4 Molecular docking

Molecular docking analysis provide valuable *in silico* support for the observed bioactivities of NJEO by identifying potential interactions between its key phytoconstituents and selected target proteins (*L. sanfranciscensis* Nox, *S. aureus* TyrRS, and *E. coli* FabB). Notably, the consistent high-affinity binding predicted for compound 15 across all three targets suggests its role as a potential multi-target effector within the complex mixture, offering a computational rationale for the broad-spectrum efficacy indicated by our bioassays.

When interpreted within the broader context of *N. jatamansi* research, these computational findings introduce a mechainstic dimension often absent from prior studies, which have primarily focused on compositional or phenotypic reporting. While previous literature has established the antimicrobial and antioxidant profiles of the EO, our docking results propose specific molecular targets and interaction modes-such as the potential inhibition of *S. aureus* TyrRS-that could underlie these observed effects.

However, these interpretations must be tempered with appropriate caution. Molecular docking remains a predictive tool with inherent limitations. High docking scores, while indicative of favorable binding, do not equate to confirmed biological activity. Factors such as solvation effects, protein flexibility, and the entropic contributions to binding are only partially accounted for in such simulations. Moreover, the synergistic or antagonistic interactions among multiple oil constituents in a physiological setting cannot be fully captured by studying isolated compound *in silico*.

Thus, while the docking results meaningfully hypothesize that compounds such as 15, 4, and 22 may contribute to NJEO’s bioactivity through interactions with key enzymatic targets, they should be viewed as the starting point for experimental validation—for instance, through *in vitro* enzyme inhibition assays or mutational studies of the identified residue contacts. This integrated approach, combining computation with validation, would significantly strengthen the mechanistic claims and enhance the translational relevance of the findings.

## 5 Conclusion

In conclusion, this study provides a comprehensive analysis of NJEO, establishing a clear correlation between its specific chemical composition and its pronounced *in vitro* bioactivities. The chemical profiling via GC–MS identified 30 compounds, forming the material basis for its observed functions. NJEO demonstrated significant antioxidant capacity and selective antibacterial efficacy, particularly against Gram-positive *S. aureus*, which provides modern scientific support for its traditional ethnopharmacological uses. Notably, the molecular docking results offer, for the first time, a theoretical mechanistic framework at the molecular level, suggesting that the broad-spectrum activity may stem from multi-target interactions of key constituents such as compound 15. This integrated phytochemical, bioactivity, and computational approach significantly advances the understanding of NJEO’s functional basis.

Howerer, it is crucial to acknowledge that the current findings are derived from *in vitro* assays and computational predictions. To translate this potential into practical applications, future research should focus on: (1) comparing the chemical and activity profiles of *N. jatamansi* from different geographical origins or chemotypes to identify optimal sources; (2) progressing to *in vivo* studies to confirm efficacy and safety within complex biological systems; (3) isolating and purifying key active compounds (e.g., compound 15) to validate their individual contributions and synergistic effects; and (4) experimentally verifying the predicted protein-ligand interactions through techniques such as enzymatic inhibition assays. These steps are essential for substantiating the mechanisms proposed herein and for laying a robust foundation for the development of NJEO in fields such as natural food preservatives or complementary antimicrobial agents.

## Data Availability

The original contributions presented in the study are included in the article/Supplementary material, further inquiries can be directed to the corresponding authors.

## Supplementary material

Supplementary Figure 1 Antibacterial activity of NJEO and positive controls against *S. aureus* and *E. coli*.
