# Supplementary figures and images for: Chemical analysis, antioxidant and antimicrobial activities of Nardostachys jatamansi essential oil, and computational evaluation of mechanisms
Source: Front Nutr. 2026 Mar 3;13:1764021. doi: 10.3389/fnut.2026.1764021 (PMC12992287; doi:10.3389/fnut.2026.1764021)

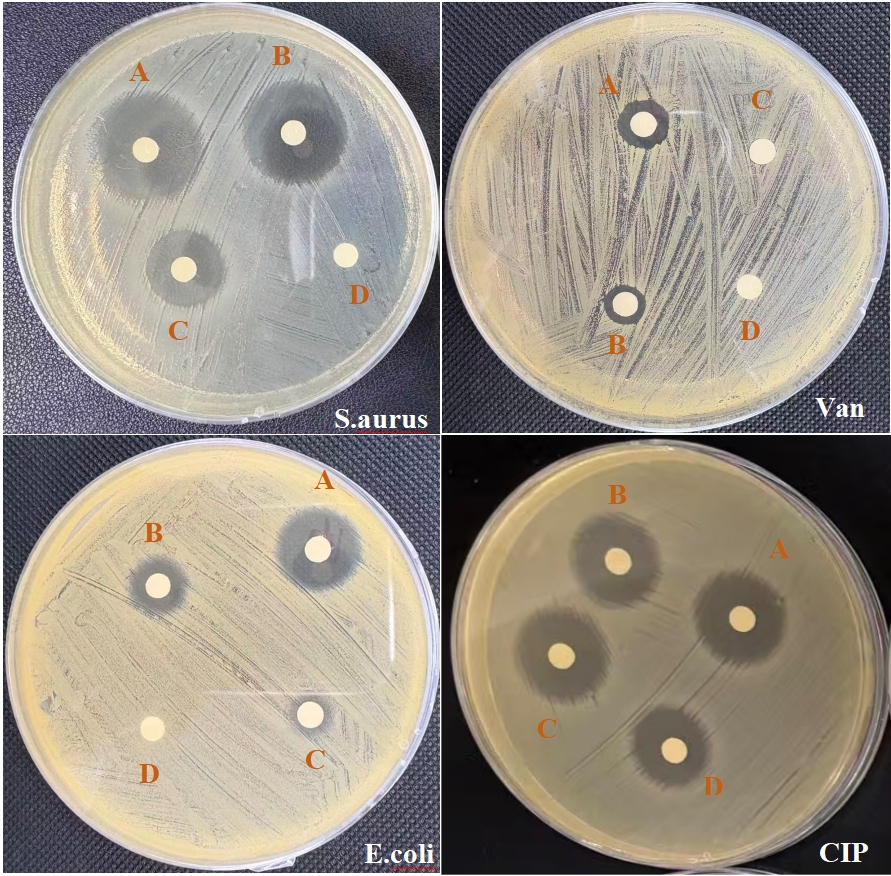

Supplement: Supplementary Figure 1 — Antibacterial activity of NJEO and positive controls against S. aureus and E. coli. [file Image_1.JPEG]
